# Novel Compound Heterozygous Variants in the *COG5* Gene Causing Fetal Hydrops and Skeletal Dysplasia

**DOI:** 10.1002/mgg3.70215

**Published:** 2026-04-08

**Authors:** Qi Yang, Wei He, Qiang Zhang, Sheng Yi, Xunzhao Zhou, Linlin Wang, Shang Yi, Zailong Qin, Jingsi Luo

**Affiliations:** ^1^ Guangxi Key Laboratory of Birth Defects Research and Prevention Guangxi Key Laboratory of Reproductive Health and Birth Defects Prevention, Maternal and Child Health Hospital of Guangxi Zhuang Autonomous Region Nanning China; ^2^ Department of Genetic and Metabolic Central Laboratory Maternal and Child Health Hospital of Guangxi Zhuang Autonomous Region Nanning China; ^3^ Guangxi Clinical Research Center for Birth Defects Maternal and Child Health Hospital of Guangxi Zhuang Autonomous Region Nanning China; ^4^ Prenatal Diagnosis Center Maternal and Child Health Hospital of Guangxi Zhuang Autonomous Region Nanning People's Republic of China; ^5^ Guangxi Clinical Research Center for Pediatric Diseases Maternal and Child Health Hospital of Guangxi Zhuang Autonomous Region Nanning China

**Keywords:** *COG5*, COG5‐CDG, novel variant, prenatal ultrasound, whole‐exome sequencing

## Abstract

**Introduction:**

Congenital Disorders of Glycosylation (CDG) are a complex and highly heterogeneous group of rare metabolic disorders characterized by defects in enzymes and transporter proteins crucial for glycosylation pathways, including N‐linked, O‐linked, and glycolipid glycosylation. To date, over 160 distinct subtypes have been identified. CDG are characterized by significant clinical heterogeneity, which presents substantial challenges for diagnosis, especially in the prenatal setting. While prenatal ultrasound serves as a crucial tool for screening potential cases, the intricate nature of CDG often necessitates the use of advanced techniques such as whole‐exome sequencing (WES) to obtain more precise molecular genetic evidence.

**Methods:**

To investigate the genetic causes of suspected CDG in a Chinese fetus, WES was performed. Subsequently, the detected variants and their origins were validated by Sanger sequencing and classified according to the guidelines of the American College of Medical Genetics and Genomics/Association for Molecular Pathology (ACMG/AMP).

**Results:**

A non‐consanguineous couple had previously experienced fetal hydrops with skeletal dysplasia during midpregnancy. In their subsequent pregnancy, second‐trimester ultrasonography again revealed fetal hydrops, a previously unreported complication. Fetal WES revealed two novel heterozygous variants in the *COG5* gene (NM_006348.5), namely c.1972del(p.Val658Serfs*23) and c.2168_2168+4delinsCATAAAA. Sanger sequencing confirmed that the c.1972del(p.Val658Serfs*23) variant was paternally inherited, while the c.2168_2168+4delinsCATAAAA variant was maternal inherited. Both variants were classified as likely pathogenic according to the ACMG/AMP guidelines.

**Conclusions:**

This study further expands the phenotypic and mutational spectrum of the *COG5* gene, enhancing our understanding of COG5‐CDG. Clinically, intellectual disability, developmental delay, brain abnormalities, skeletal deformities, microcephaly, short stature, vision abnormalities, and hepatic lesions are crucial diagnostic criteria for COG5‐CDG. Other variable phenotypes of COG5‐CDG can provide supporting information for prenatal diagnosis. The combined application of prenatal ultrasound and WES enables a more comprehensive and precise diagnosis of COG5‐CDG, which will facilitate the implementation of early intervention and treatment.

## Graphical abstract

Two affected fetuses in a COG5‐CDG family exhibited fetal hydrops and skeletal malformations, which were found to segregate with the paternal frameshift variant c.1972del and the maternal splice‐site variant c.2168_2168+4delinsCATAAAA in the *COG5* gene.

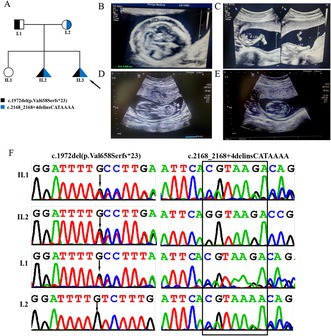

## 1 Introduction

Congenital disorders of glycosylation (CDGs) are a highly heterogeneous group of metabolic genetic disorders caused by defects in protein glycosylation (Ondruskova et al. 2021). Patients with CDG present with a wide range of clinical manifestations, often involving multiple systems, including neurological manifestations, dysmorphic features, growth retardation, musculoskeletal abnormalities, hepatic lesions, coagulopathies, recurrent infections, and endocrine deficiency (Marques‐da‐Silva et al. 2017; Péanne et al. 2018). These symptoms may be present from birth. To date, over 160 distinct CDG subtypes have been identified, collectively implicating defects in enzymes and trafficking factors critical to N‐linked, O‐linked, and glycolipid glycosylation pathways (Francisco et al. 2019; Ferreira et al. 2018).

Within this spectrum, the conserved oligomeric Golgi (COG) complex is a critical Golgi‐associated tethering factor that regulates vesicular trafficking and glycoprotein modification (Willett et al. 2013; Blackburn et al. 2019). This octameric complex comprises two functionally interdependent lobes: lobe A (COG1‐4) and lobe B (COG5‐8) (Fotso et al. 2005; Ungar et al. 2005). Pathogenic variants in COG1, COG2, COG4, COG5, COG6, COG7, or COG8 disrupt complex integrity, causing abnormal glycosylation of glycoproteins and glycolipids. This molecular dysfunction manifests clinically as multi‐organ system pathology, spanning severe infantile presentations to moderate neurological impairment phenotypes (Ungar et al. 2002; Barone et al. 2014). As a critical subunit of the COG complex, COG5 plays essential roles in Golgi trafficking, glycosylation fidelity, and structural maintenance of the complex (Blackburn et al. 2019). Deficiencies in COG5 result in COG5‐CDG, a specific subtype of CDG (Paesold‐Burda et al. 2009). To date, only 16 cases have been documented worldwide (Table 1) (Paesold‐Burda et al. 2009; Fung et al. 2012; Rymen et al. 2012; Chérot et al. 2018; Yin et al. 2019; Ferrer et al. 2020; Tabbarah et al. 2020; Buyukdogan et al. 2023; Wang et al. 2020, 2024). The pathophysiological mechanisms linking distinct *COG5* variants to their heterogeneous clinical manifestations remain incompletely understood. Additional reports characterizing *COG5* variants and their corresponding phenotypic spectra will be crucial for advancing disease comprehension and elucidating genotype–phenotype correlations. Here, we report two cases of Chinese fetuses from the same family with novel compound heterozygous variants in the *COG5* gene. We also describe the associated clinical features of the fetuses, thereby expanding the mutation and phenotypic spectrum of COG5‐CDG.

**TABLE 1:** Summary of reported information on clinical phenotypes in association with *COG5* variants in patients with COG5‐CDG.

P	Gender	Age at last examination	*COG5* variants (NM_006348)	*ID/DD*	Hypotonia	Brain abnormalities	Microcephaly	Vision abnormalities	Neurogenic bladder	Ataxia	Liver lesion	Short stature	Deafness	Skeletal abnormality	Other features	References
1	NA	17‐week‐old fetus	c.1972del(p.V658Sfs*23); c.2168_2168+4delinsCATAAAA	NA	NA	NA	−	NA	NA	NA	NA	NA	NA	NA	Fetal hydrops	This study
2	NA	14‐week‐old fetus		NA	NA	NA	−	NA	NA	NA	NA	NA	NA	+	Fetal hydrops	
3	F	4Y	c.2077A>C(p.T693P); c.1290C>A(p.Y430X)	+	−	−	−	+	−	+	+	−	−	+	Hypohidrosis, hyperkeratosis; coagulation defect	Wang et al. (2020)
4	F	8Y	c.1669‐15A>G	+	+	+	−	+	−	+	−	−	−	−	−	Paesold‐Burda et al. (2009)
5	F	9Y	c.556_560delAGTAAinsCT; c.1919 T>C(p.I640T)	+	+	+	+	−	−	−	+	+	−	+	Mild thrombocytopenia; persistent mild hyperlactacidemia; portal hypertension	Fung et al. (2012)
6	F	1Y	c.2518G>T(p.E840X)	+	+	+	+	−	−	−	−	+	−	−	−	Rymen et al. (2012)
7	F	N/A	c.2518G>T(p.E840X)	+	+	N/A	+	−	+	+	−	+	−	−	Autistic behavior	
8	F	1 M	c.556_560delAGTAAinsCT; c.95T>G(p.M32R)	+	+	+	+	−	−	−	+	−	−	+	Mild thrombocytopenia; persistent mild hyperlactacidemia; portal hypertension.	
9	F	8 M	NA	+	+	−	+	+	+	−	−	+	−	−	Autistic behavior	
10	M	3 M	c.189delG(p.C64Vfs*6); c.2338_2340dupATT(p.I780dup)	+	+	+	+	+	+	NA	+	−	+	+	Recurrent urinary tract infection; spastic quadriplegia	
11	M	At birth	c.1780G>T(p.V594F)	+	+	−	+	+	+	NA	−	+	+	+	Hypohidrosis, epilepsy; micropenis with cryptorchidism.	
12	M	11Y	c.330delT(p.V111Lfs*22); c.2324 C>T(p.P775L)	+	+	+	+	−	−	−	+	−	−	+	Convulsions	Yin et al. (2019)
13	F	11Y	c2T>G(P.M1?), c1826T>C(p.I608V); c.556–560delAGTAAinsCT	+	+	−	−	+	−	−	+	−	−	+	Mild inverted nipples	Wang et al. (2024)
14	F	21Y	c.95T>G(p.M32R); c.2327dup(S777Efs*14)	−	NA	+	+	+	NA	−	NA	+	−	+	−	Tabbarah et al. (2020)
15	F	NA		−	NA	+	+	+	NA	−	NA	+	+	+	−	
16	M	18Y		−	NA	+	+	+	NA	−	NA	+	−	+	−	
17	NA	30‐week‐old fetus	c.944C>G(p.Ser315Cys)	NA	NA	+	−	NA	NA	NA	NA	+	NA	+	congenital heart defect, dysmorphic features, other variant (ALG3 c.1188G>A, p.Trp396*)	Ferrer et al. (2020)
18	NA	24‐week‐old fetus	c.95T>G(p.Met32Arg)	NA	NA	+	−	NA	NA	NA	NA	+	NA	+	Polyhydramnios, abnormal kidney morphology, dysmorphic features	Buyukdogan et al. 2023
Total = 18				11/14	10/11	12/15	11/18	9/14	4/11	3/12	6/11	10/16	3/15	13/17		

Abbreviations: DD, developmental delay; F, female; ID, intellectual disability; M, male; M, months; NA, not available; P, patient; Y, years.

## 2 Material and Methods

### 2.1 Subjects and Ethics Approval

The study was approved by the Institutional Review Board and Ethics Committee of Guangxi Maternal and Child Health Hospital, and the patients' parents provided detailed informed consent for the publication of the relevant clinical data.

### 2.2 Prenatal Ultrasonography

Ultrasound screening of pregnant women was done with a 3–6 MHz transabdominal probe on the Toshiba Aplio500 color Doppler ultrasonic diagnostic device (Toshiba, Tokyo, Japan). The settings adhered to the manufacturer's suggested specifications. A senior fetal ultrasound‐trained physician (5+ years of experience) conducted the screening. Two consultant physicians took the measurements, which were verified thrice by the same examiner for precision. The average of these readings was recorded as the final result.

### 2.3 Genetic Analysis

Fetal DNA was extracted from chorionic villus samples using the QIAamp DNA Mini Kit (Qiagen, Germany). Genomic DNA from peripheral blood lymphocytes of all subjects was isolated with the La‐Aid DNA kit (Zeesan Biotech Co. Ltd., Xiamen, China). For whole exome sequencing (WES), exomic libraries were constructed from genomic DNA using the Agilent SureSelect Human All Exon V6 Kit (Agilent Technologies, Santa Clara, CA), following the manufacturer's protocol. The libraries were sequenced on the Illumina HiSeq2500 platform (Illumina, San Diego, CA). The generated reads were aligned to the human reference genome (hg19/GRCh37) using the BWA package (v. 0.7.15). Variant calling and annotation were performed with the TGex software (LifeMap Sciences Inc., v5.7) and the Genome Analysis Toolkit (GATK). The pathogenicity of candidate variants was predicted and prioritized using Mutation Taster, CADD, SIFT, PROVEAN, and PolyPhen 2.0. Sanger sequencing was used to validate the variant in the fetal and unaffected family members. Classification and interpretation of identified variants adhered to the guidelines of the American College of Medical Genetics and Genomics (ACMG)/Association for Molecular Pathology(AMP) (Richards et al. 2015; Miller et al. 2023).

## 3 Results

### 3.1 Clinical Phenotype

**FIGURE 1:**
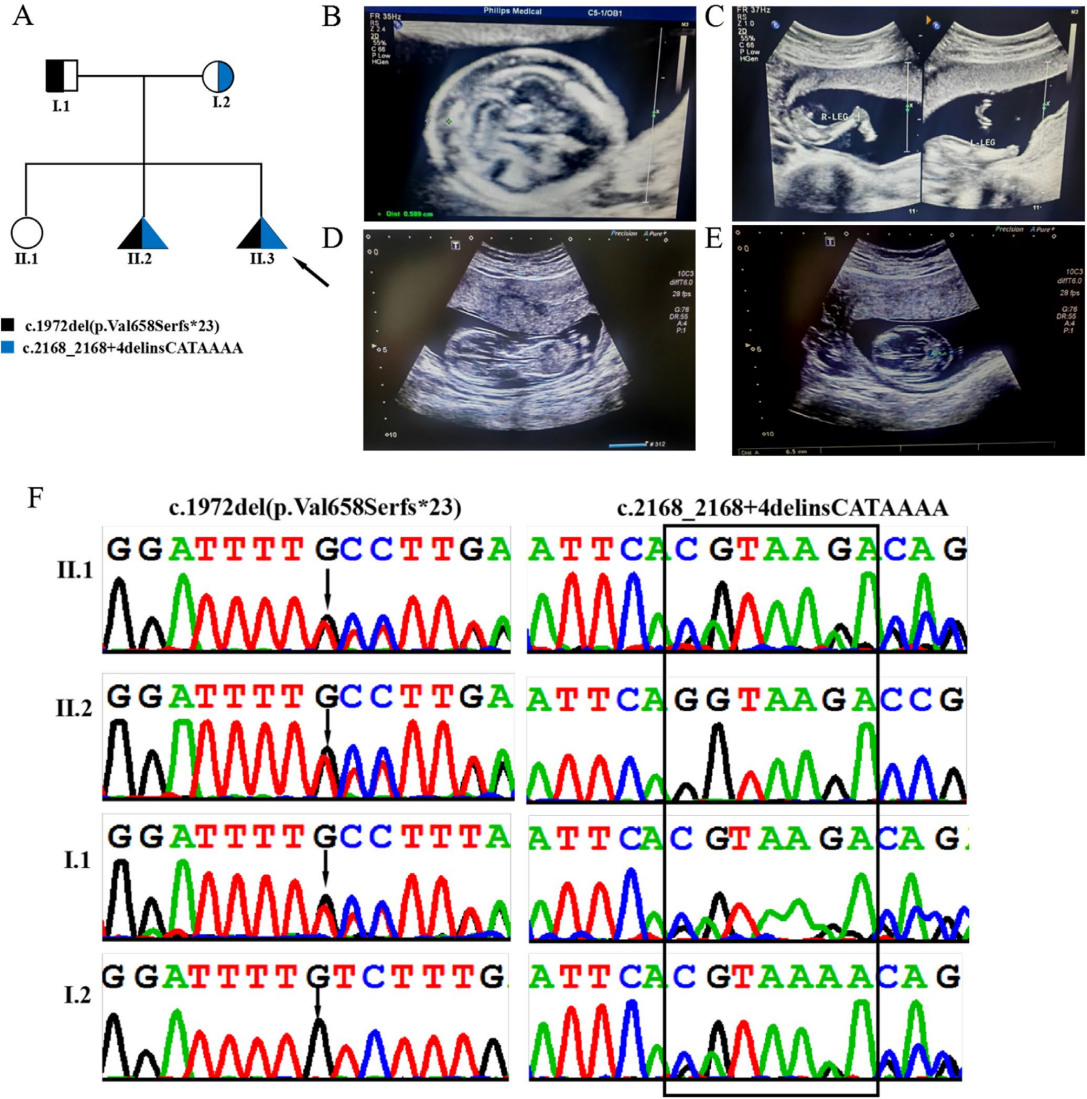
Clinical and genetic features. (A) Pedigree chart of a family with two fetuses affected by COG5‐CDG, in which the proband is indicated by the black arrow. (B) Fetal hydrops was detected in fetus II.2 via ultrasound at 14 weeks of gestation. (C) At 17 weeks gestation, ultrasound revealed skeletal abnormalities in the fetus (II.2), including extension of the left knee, flexion of the right knee, and bilateral rocker‐bottom foot. (D, E) At 14 weeks of gestation, an ultrasound scan revealed fetal hydrops in Fetal II.3. (F) Sanger sequencing DNA chromatograms of COG5 indicating the frameshift variant c.1972del(p.Val658Serfs*23) inherited from the father and the splicing variant c.2168_2168+4delinsCATAAAA was transmitted by the mother.

A 32‐year‐old woman, gravida 3 para 1 (G3P1), was referred to the Genetics Department at 14 weeks and 4 days of gestation (Figure 1A). She had a history of pregnancy termination due to generalized fetal hydrops and skeletal abnormalities. During that pregnancy at 17 weeks, prenatal ultrasound revealed fetal skeletal abnormalities manifested by extension of the left knee, flexion of the right knee, and bilateral rocker‐bottom feet (Figure 1B,C). Chromosomal microarray analysis (CMA) detected no pathogenic copy number variants (deletions/duplications). The couple are non‐consanguineous and have a healthy 3‐year‐old daughter. The current pregnancy was initially unremarkable. The patient denied any maternal medical conditions or exposure to teratogens. At 14 weeks and 4 days of gestation, ultrasound demonstrated generalized hydrops (Figure 1D,E). The pregnant woman opted to terminate the pregnancy at 15 weeks of gestation; no other abnormalities were detected in the postoperative examination.

### 3.2 Genetic Analysis

Whole‐exome sequencing (WES) was performed to investigate fetal genetic variants. The analysis yielded 8.46 Gb of data, achieving 99.3% target region coverage with 99.2% of regions covered at ≥ 20 × depth. The initial screen detected 111,003 SNVs/indels in coding regions and splice sites (±10 bp). After filtering out variants with MAF > 1% via population databases (gnomAD, dbSNP132, ESP, 1000G, in‐house), 819 unique SNPs remained. Further exclusion of predicted benign variants (including synonymous variants and missense variants predicted to be harmless by in silico prediction tools) left 543 candidates. Following data analysis using the TGex analysis software (https://tgex.genecards.cn/), seven variants were extracted from six genes (*CELSR1*, *COG5*, *COL1A1*, *SLC32A1*, *DNM1*, *VSX1*) which matched known phenotypes. Variants in *CELSR1*, *COL1A1*, *SLC32A1*, *DNM1*, and *VSX1* were inherited from unaffected parents, excluding pathogenicity. Two heterozygous *COG5* variants were ultimately identified, namely c.1972del(p.Val658Serfs*23) and c.2168_2168+4delinsCATAAAA. Sanger sequencing confirmed paternal inheritance of c.1972del(p.Val658Serfs*23) and maternal transmission of c.2168_2168+4delinsCATAAAA (Figure 1F).

## 4 Discussion

In this study, we describe an additional Chinese family with two affected fetuses presenting fetal hydrops, thickened nuchal fold and skeletal deformities. These phenotypes were caused by a novel compound heterozygous variant in the *COG5* gene, specifically c.1972del (p.Val658Serfs*23) and c.2168_2168+4delinsCATAAAA. Sanger sequencing confirmed that the variants c.1972del(p.Val658Serfs*23) and c.2168_2168+4delinsCATAAAA were inherited from the parents, respectively. Both variants were not found in the Human Gene Mutation Database (http://www. hgmd.cf.ac.uk/ac/), HPSD (http://liweilab.genetics. ac.cn/HPSD/), dbSNP (http://www.ncbi.nlm.nih.gov/SNP/), ExAC, and gnomAD (https://gnomad.broad institute.org/). The c.1972del(p.Val658Serfs*23) variant is located in the eighteenth exon of the *COG5* gene and causes a premature termination codon, resulting in loss of function. MutationTaster predicted that c.1972del(p.Val658Serfs*23) is disease‐causing. The other *COG5* variant, c.2168_2168+4delinsCATAAAA, is a splicing variant. This variant alters the classical splicing donor site (+1 to +5), predicted to disrupt the native GT dinucleotide sequence. In silico tools (Splice‐AI Δ‐score = 0.95, Human Splicing Finder “5′ donor disrupted”) uniformly indicate complete loss of the wild‐type splice site, and no plausible cryptic site is created. It is predicted to cause exon 19 skipping, resulting in the deletion of 77 base pairs and a frameshift mutation. This significantly reduces protein production and markedly reduces mRNA levels, leading to a loss of function. According to the criteria and guidelines of the ACMG/AMP for the interpretation of sequence variants, c.1972del(p.Val658Serfs*23) was assessed to be pathogenic based on PVS1 (null variant in a gene with established loss‐of‐function mechanism), PM2_Supporting (absent from population databases), and PP1 (co‐segregation with disease in multiple affected family members); and c.2168_2168+4delinsCATAAAA was assessed to be pathogenic based on PVS1 (predicted to result in null variant through exon skipping), PM2_Supporting, PM3 (for recessive disorders, detected in trans with a pathogenic variant), and PP1 (co‐segregation with disease in multiple affected family members) (Table 2).

**TABLE 2:** Predicted pathogenicity of novel *COG5* variants.

Gene	Variant (NM_006348)	Inheritance	LRT	MutationTaster	Splice‐AI	NMDEscPredictor	ACMG/AMP
*COG5*	c.1972del(p.V658Sfs*23)	Paternal	D	D	NA	NMD	P[(PVS1 + PM2_Supporting+PP1)]
*COG5*	c.2168_2168+4delinsCATAAAA	Maternal	NA	D	0.95	NMD	P[(PVS1 + PM2_Supporting+PM3 + PP1)]

Abbreviations: D, deleterious or damaging; NA, not available; NMD, nonsense mediated decay; P, pathogenic.

The medical symptoms and genetic information of patients (including those from the present study) with COG5‐CDG are shown in Table 1. Only 18 affected individuals from 12 unrelated families (including our patients) have been reported to carry *COG5* gene variants worldwide (Table 1). Despite the highly heterogeneous clinical manifestations of these cases, we still observed some relatively consistent phenotypes. Almost all patients (11/14) exhibited intellectual disability and/or developmental delay. Notably, over half of the patients presented with moderate to severe intellectual disability and language developmental delay. These patients also exhibited hypotonia (10/11), which improved with age, and all of them learned to walk, although some had gait abnormalities. Brain abnormalities are also observed in most patients (12/15) and can first appear during the fetal period. For example, a 24‐week‐old fetus was observed to have severe brain abnormalities. These abnormalities include diffuse atrophy of the cerebellum and brainstem, delayed myelination, overall reduction of white matter, as well as enlarged lateral ventricles and severe atrophy of the cerebral hemispheres and cerebellum. Skeletal and limb abnormalities, including camptodactyly and clinodactyly, flexion contractures, slightly smaller feet, spina bifida, vertebral column abnormality, scoliosis, and micrognathia, are common (13/17). Among these patients, three fetuses had skeletal deformities detected by ultrasound examination during the fetal period. This early finding strongly suggests that abnormalities in the skeletal system may be a typical and prominent feature of the disease during the fetal period. This makes prenatal ultrasound an important tool for early identification and diagnosis, and it has significant guiding significance for genetic counseling and perinatal management. Microcephaly (11/18), short stature (10/16), visual abnormalities (such as strabismus and cortical blindness) (6/11), and hepatic lesions (6/11) are also common. Other variable phenotypes were also observed, such as neurogenic bladder, ataxia, deafness, epileptic seizures, hypohidrosis, hyperkeratosis, coagulation defects, hyperlactatemia, and autistic behaviors.

To the best of our knowledge, this is the third report of fetal glycosylation disorders caused by variants in the *COG5* gene. Similar to the two previously reported fetuses, fetus 1 in this study also exhibited skeletal dysplasia (Ferrer et al. 2020; Buyukdogan et al. 2023). Although no skeletal malformations were observed in fetus 2 (II 3) at 14 weeks and 4 days of gestation, the possibility of other congenital anomalies developing with advancing pregnancy could not be excluded. However, the patient elected to terminate the pregnancy at 16 weeks, and postoperative examination revealed no such abnormalities. In contrast to the two previously reported fetuses, neither of the two fetuses in this study was observed to have severe brain malformations or facial dysmorphic features. Meanwhile, fetal hydrops, a new symptom, was observed in both fetuses in this study. Fetal hydrops is a common clinical feature of CDG. It is worth noting that among CDG cases, 17 patients carrying variants in the *PMM2*, *ALG1*, *ALG8*, *ALG9*, or *MGAT2* genes presented with fetal hydrops due to N‐glycosylation defects, and 1 patient carrying a variant in the COG6 gene presented with fetal hydrops due to multiple glycosylation defects involving both N‐ and O‐glycosylation pathways (Helenius and Aebi 2001; Makhamreh et al. 2020). COG5 is involved in the N‐glycosylation pathway, which may be the cause of fetal hydrops in these two cases (Altassan et al. 2019; Fisher and Ungar 2016; Ioffe and Stanley 1994). These results further emphasize the complexity and diversity of the COG5‐CDG phenotype. Detailed clinical descriptions and additional case reports will enhance our understanding of this disease. Moreover, the results of this study indicate that WES combined with ultrasound examination has significant application value in the prenatal diagnosis of fetal malformations. This combined diagnostic model can improve the accuracy and detection rate of fetal malformations, provide more comprehensive and accurate diagnostic information for clinicians and families, and help better assess the health status of the fetus and make more reasonable prenatal decisions.

This study has several limitations. Due to the limited number of reported cases and variant spectrum, the phenotypic profile still needs to be expanded. As the number of patients increases, genotype–phenotype correlations and potential modifying factors are expected to be further clarified. This study did not include a direct evaluation of the functional implications of the variants. Therefore, additional functional investigations are required, including in vitro splicing reporter assays, protein expression analysis and cellular models. These will enhance our understanding of COG5‐CDG and its mechanisms of action.

In conclusion, our study has identified a novel compound heterozygous variant of the *COG5* gene in two fetuses from the same family diagnosed with COG5‐CDG. These variants are associated with fetal hydrops and skeletal abnormalities. These findings further expand the COG5‐CDG phenotypic and variant spectrum. Moreover, the detailed characterization of the patients' diverse phenotypes deepens our understanding of the COG5‐CDG phenotypic spectrum. This comprehensive phenotypic analysis is crucial for precise clinical diagnosis and effective genetic counseling. Furthermore, this study demonstrates that integrating WES with ultrasound examination enables early, precise prenatal diagnosis. This combined approach empowers clinicians to make informed decisions and implement appropriate management strategies in high‐risk pregnancies. We recommend adopting such combined diagnostic approaches in clinical practice to improve outcomes for families at risk of CDG and other inherited disorders.

## Author Contributions

Q.Y. and J.L. designed and drafted the manuscript. W.H., Q.Z., S.Y., X.Z., S.Y., L.W. and Z.Q. collected the patients' clinical information and analysed the WES data. Q.Y., W.H., Q.Z. and J.L. revised the manuscript. All authors contributed to the coordination of the study and revised the manuscript. All authors read and approved the final version of the manuscript.

## Funding

This research was supported by the Guangxi Science and Technology Program (21–220‐22 and GuiKe LT2600640039), the Guangxi Natural Science Foundation (Grant No. 2026GXNSFAA00641253), and the Health Department of Guangxi Province (Grant No. Z‐A20220256).

## Ethics Statement

This study was approved by the Institutional Review Board and Ethics Committee of Guangxi Maternal and Child Health Hospital and conducted in accordance with the principles of the Declaration of Helsinki. All procedures followed relevant guidelines and regulations. Written informed consent for the publication of potentially identifiable images or data was obtained from the patients or their legal guardians.

## Conflicts of Interest

The authors declare no conflicts of interest.

## Data Availability

The data that support the findings of this study are available on request from the corresponding author. The data are not publicly available due to privacy or ethical restrictions.
